# Settlement on asphalt concrete pavement in permafrost regions under the dynamic load of aircraft

**DOI:** 10.1371/journal.pone.0288785

**Published:** 2023-07-21

**Authors:** Xiaolan Liu, Yixiang Chen, Chuanwei Fu

**Affiliations:** Tianjin Key Laboratory of Civil Structure Protection and Reinforcement, Tianjin Chengjian University, Tianjin, China; Shandong University of Technology, CHINA

## Abstract

The settlement in permafrost regions has a significant effect on the safety of the aircraft. Therefore, a numerical model of asphalt concrete pavement and subgrade in permafrost regions is proposed and verified by comparing with previous studies. Numerical models under the dynamic load of aircraft are used to analyze the settlement nephogram, time-dependent curve, and settlement curve. Results show that the influences of different parameters on settlements decrease slowly at the depth of 0–1.45m, then decrease quickly at the depth of 1.45-2m, finally tend to be stable. The peaks of settlements increase with the growth of international roughness index, taxiing speed, and aircraft’s weight. The settlement increases little when the international roughness index is more than 3. The settlement varies significantly when the taxiing speed is from 30m/s to 70m/s. The study provides a theoretical basis for the construction and maintenance of asphalt concrete pavement and subgrade in permafrost.

## 1. Introduction

A modern international corridor of the airport system oriented to "Belt and Road" will be gradually built to promote the connectivity between China and its surrounding countries in the future [1]. Hence, 68 new and renovated general airports will be built in permafrost regions in accordance with the medium-term and long-term plan for airport construction in China [1]. However, the permafrost is thermally unstable, and is easy to cause the thawing and settlement with the effect of pavement weights and aircraft loads [2]. Moreover, the investigation results show that engineering structures in permafrost regions suffer from cracks, deformation, collapse caused by settlement. Therefore, it is urgent to settle the matter of settlement in the construction of airport in a permafrost region affected by the temperature, pavement weights, and aircraft loads.

Scholars had studied the settlement of permafrost [3, 4]. Morgenstern and Nixon [5], and Foriero and Ladanyi [6] applied Terzaghi consolidation theory, the large strain thawing consolidation theory, and heat conduction equation to analyze the settlement of the saturated and unsaturated soil. Hou [7] adopted conservation law of momentum and mass, entropy inequality, and the mixture theory to established the settlement model. They considered the influence of the volume fraction condition. With the development of computer technology, Fortier et al. [8], and Wang [9] applied the water-thermal-mechanical coupling model to analyze the influence of subgrade parameters, snow cover, slope orientation on the settlement. Their study showed that the thawing inter-layer, slope effect, and thawing depth had the significant impact on the uneven settlement. Liu [10], and Zhao [11] explored the influence of subgrade parameters and freeze-thaw cycles on the mechanism of railway settlement based on the water-heat-mechanical response model. Vladimir and Elena [12] had successfully predicted the response of settlement in the Russian northern ports based on the numerical simulation method. In addition, scholars had also carried out a large number of experimental studies. Zaretskii et al. [13], Arenson et al. [14], Zheng [15], and Kenan [16] analyzed the connection of settlement, dry density, moisture content, and freezing temperature based on the triaxial compression test, volume change test, unconfined compression test, and freeze-thaw cycle test. Peng et al. [17], Zhu [18], and Lu et al. [19] used the in-situ data of Qinghai-Tibet highway to conclude four types of settlement patterns, including low steady subsidence, fluctuating subsidence, step subsidence and high steady subsidence. Their research explained that the wave of pavement, and the longitudinal crack and collapse of subgrade mainly occurred in the first freeze-thaw cycle stage and were caused by the longitudinal and lateral settlement. Ma [20] and Chen [21] studied the evolution law of settlement using the in-situ monitoring data from the Qinghai-Tibet Highway test. Wang [22] explored the mechanism of thawing settlement of tunnel in a permafrost region, and discovered that the thawing settlement primarily took place in the early stage. Dong [23] and Wang et al. [24] analyzed the mechanism of settlement of pipeline by the field trial.

The above research focuses on railway, highway, tunnel, and pipeline engineering, and does not fully considered the characteristics of pavement in the airport including wide width, short length, low pavement roughness, and large aircraft’s weight. Meanwhile, the existing research on the mechanism of settlement has more consideration on static factors including subgrade parameters, slope, and freeze-thaw cycle, but less consideration on the dynamic load of aircraft. However, the settlement of asphalt concrete pavement in permafrost regions is affected by multi-dimensional factors, such as self-weight stress from subgrade and pavement structures, subsidiary stress from dynamic load of aircraft, and temperature field from environmental factors. Moreover, the dynamic load of aircraft is affected by the aircraft’s weight, international roughness index, and taxiing speed. Therefore, based on the characteristics of airport and the actual conditions of permafrost subgrade in China, this paper establishes aircraft and pavement response model to reveal the settlement mechanism of permafrost subgrade under the dynamic load of aircraft. The study provides a theoretical basis for the construction and maintenance of asphalt concrete pavement in a permafrost region.

## 2. Theoretical analysis

### 2.1 Heat conduction equations

Because the asphalt concrete pavement is disturbed by solar precipitation and radiation, water content of structural layer and geological lithology, some assumptions are made to ensure computational efficiency and effectiveness. Firstly, the pavement, natural surface, and subgrade are uniform and isotropic. Secondly, the boundary of pavement, natural surface, and subgrade does not consider the migration of moisture. Thirdly, the layers of pavement, natural surface, and subgrade meet the law of energy conservation, and only include the effects of heat transfer. Therefore, the three-dimensional unsteady heat conduction express as Eq (1) during freezing and thawing [25, 26]:

$$C\frac{\partial T}{\partial t}-L\rho_{i}\frac{\partial w_{i}}{\partial t}=\frac{\partial }{\partial x}(k_{x}\frac{\partial T}{\partial x})+\frac{\partial }{\partial y}(k_{y}\frac{\partial T}{\partial y})+\frac{\partial }{\partial z}(k_{z}\frac{\partial T}{\partial z})$$ (1)

where *C* and *T* are the heat capacity and the temperature, respectively. *t* and *L* are the time and the transformed latent heat of water, respectively. *ρ*_*i*_ and *w*_*i*_ are the density and volume fraction of ice, respectively. *k*_*x*_, *k*_*y*_, and *k*_*z*_ are the components of thermal conductivity.

Meanwhile, the three-dimensional mass transfer express as Eq (2) with evaporation and other factors being ignored [26, 27]:

$$\frac{\partial w_{u}}{\partial t}+\frac{\rho_{i}}{\rho_{w}}\frac{\partial w_{i}}{\partial t}=\frac{\partial }{\partial x}(K_{x}\frac{\partial \varphi }{\partial x})+\frac{\partial }{\partial y}(K_{y}\frac{\partial \varphi }{\partial y})+\frac{\partial }{\partial z}(K_{z}\frac{\partial \varphi }{\partial z})$$ (2)

where *w*_*u*_ and *ρ*_*w*_ are the volume fraction of unfrozen water and the density of water, respectively. *K*_*x*_, *K*_*y*_, and *K*_*z*_ are the components of hydraulic conductivity. *φ* is the total potential energy, and equals to *ϕ*+*h*. *ϕ* and *h* are the volumetric potential energy and the gravitational potential, respectively. *h* can be ignored.

The content of unfrozen water can be shown in Eq (3) [26]:

$$\frac{\partial w_{u}}{\partial t}=\frac{\partial w_{u}}{\partial T}\frac{\partial T}{\partial t}$$ (3)

Eqs (1)–(3) can applied to obtain Eq (4) [26]:

$$\left(C+L\rho_{w}\frac{\partial w_{u}}{\partial T})\frac{\partial T}{\partial t}=\frac{\partial }{\partial x}(k_{x}\frac{\partial T}{\partial x})+\frac{\partial }{\partial y}(k_{y}\frac{\partial T}{\partial y})+\frac{\partial }{\partial z}(k_{z}\frac{\partial T}{\partial z})+L\rho_{w}[\frac{\partial }{\partial x}(K_{x}\frac{\partial \phi }{\partial x})+\frac{\partial }{\partial y}(K_{y}\frac{\partial \phi }{\partial y})+\frac{\partial }{\partial z}(K_{z}\frac{\partial \phi }{\partial z})\right]$$ (4)

The volumetric potential of unfrozen water is shown in Eq (5) [26]:

$$\frac{\partial \phi }{\partial t}=\frac{\partial \phi }{\partial w_{u}}\frac{\partial w_{u}}{\partial T}$$ (5)

Eq (6) can be obtained with the differential water capacity and water diffusion coefficient into Eq (5) [26]. Based on Eqs (4) and (6), the three-dimensional unsteady heat conduction express as Eq (7) [26].

$$\frac{\partial \phi }{\partial x}=\frac{\partial \phi }{\partial T}\frac{\partial T}{\partial x}=\frac{D}{K}\frac{\partial w_{u}}{\partial T}\frac{\partial T}{\partial x}$$ (6)

$$(C+L\rho_{w}\frac{\partial w_{u}}{\partial T})\frac{\partial T}{\partial t}=\frac{\partial }{\partial x}(k_{x}+L\rho_{w}D_{x}\frac{\partial w_{u}}{\partial T})\frac{\partial T}{\partial x}+\frac{\partial }{\partial y}(k_{y}+L\rho_{w}D_{y}\frac{\partial w_{u}}{\partial T})\frac{\partial T}{\partial y}+\frac{\partial }{\partial z}(k_{z}++L\rho_{w}D_{z}\frac{\partial w_{u}}{\partial T})\frac{\partial T}{\partial z}$$ (7)

where *D* and *K* are the coefficient of water diffusion and the hydraulic conductivity, respectively.

### 2.2 Boundary conditions

Based on the surface temperature in a permafrost region, the first boundary condition is established in Eq (8) and is applied on the surface of numerical model [21, 26]:

$$T=T_{0}+\Delta T+a\sin(\frac{2\pi t}{8760}+b)+\frac{\alpha t}{8760\times 30}$$ (8)

where *T*_0_ is the mean annual ground temperature of the boundary layer, taken as the mean annual air temperature, *ΔT* is the increment in the temperature of the boundary layer, and is set to 2.5°C for the natural surface and 4.5°C for the pavement. *a* is the amplitude of variation in temperature, and is set to 12°C for the natural surfaces and 15°C for the pavement. *t* is the time in hours, *b* is the initial phase, equals to zero at the initial set time of April 1 in northeast China. *α* is the increment in the global temperature over the next 30 years, and is set to 1.5°C.

The second boundary condition is an adiabatic boundary condition in Eq (9), and is applied on both sides of numerical models [16, 26]:

$$\frac{\partial T}{\partial n}=0$$ (9)

The third boundary condition is a constant temperature gradient of 0.03°C/m. This is applied at the bottom of the numerical model, as shown in Eq (10) [26, 28]:

$$\frac{\partial T}{\partial n}=0.03$$ (10)

### 2.3 Aircraft vibration equations

**Fig 1:**
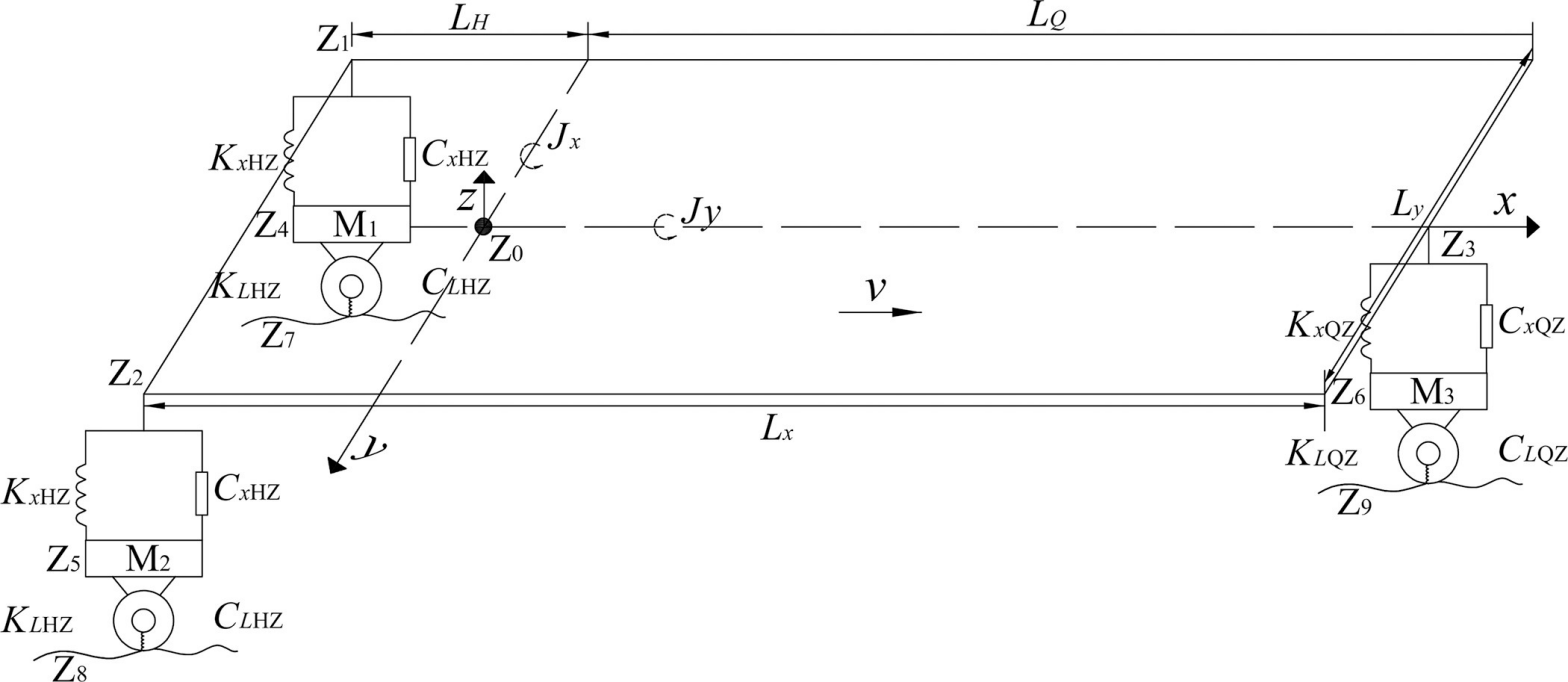
Aircraft model.

As shown in Fig 1, since the linear stiffness and damping of tires do not vary much when the aircraft is taxiing at a constant speed, it is assumed that: (1) Tires are symmetrical and all the mass is concentrated in the center of tire. (2) The circumferential deformation caused by surface friction is not considered. (3) The change of pavement roughness in the direction of tire width does not take into account. (4) The landing gear tire is composed of a plurality of spring damping units distributed along the radial direction, and the deformations are independent of each other. (5) The aircraft is considered rigid. (6) The support of landing gear is completely rigid, and the axial motion has a linear damping. (7) The dual wheels on one side are equivalent to one equivalent single wheel.

Based on the theory of time-varying mechanics, the aircraft and pavement are regarded as two independent subsystems. The contact parts between wheel and pavement are connected by the deflection coordination condition and the force balance condition. According to the vibration theory, the vibration equation of aircraft is established by combining the Lagrange equation with the force balance, moment balance and displacement coordination of system, as shown in Eqs (11)–(25) [29].

$$K_{E}=\frac{1}{2}M_{0}{\dot{Z}}_{0}^{2}+\frac{1}{2}M_{1}{\dot{Z}}_{1}^{2}+\frac{1}{2}M_{2}{\dot{Z}}_{2}^{2}+\frac{1}{2}M_{3}{\dot{Z}}_{3}^{2}+\frac{1}{2}J_{y}{\dot{\theta }}_{x}{}^{2}+\frac{1}{2}J_{x}{\dot{\theta }}_{y}{}^{2}$$ (11)

$$P_{E}=\frac{1}{2}K_{xHZ}S_{ML}^{2}+\frac{1}{2}K_{LHZ}{\left(Z_{4}‐Z_{7}\right)}^{2}+\frac{1}{2}K_{xHZ}S_{MR}^{2}+\frac{1}{2}K_{LHZ}{\left(Z_{5}‐Z_{8}\right)}^{2}+\frac{1}{2}K_{xQZ}S_{N}^{2}+\frac{1}{2}K_{LQZ}{\left(Z_{6}‐Z_{9}\right)}^{2}$$ (12)

$$D_{E}=\frac{1}{2}C_{xHZ}({\dot{S}}_{ML}^{2}+{\dot{S}}_{MR}^{2})+\frac{1}{2}C_{xQZ}{\dot{S}}_{N}^{2}$$ (13)

$$M_{0}{\ddot{Z}}_{0}+K_{xHZ}(Z_{1}-Z_{4})+C_{xHZ}({\dot{Z}}_{1}-{\dot{Z}}_{4})+K_{xHZ}(Z_{2}-Z_{5})+C_{xHZ}({\dot{Z}}_{2}-{\dot{Z}}_{5})+K_{xQZ}(Z_{3}-Z_{6})+C_{xQZ}({\dot{Z}}_{3}-{\dot{Z}}_{6})=0$$ (14)

$$J_{x}{\ddot{\theta }}_{x}+\frac{1}{2}L_{y}[K_{xHZ}(Z_{1}-Z_{4})+C_{xHZ}({\dot{Z}}_{1}-{\dot{Z}}_{4})]-\frac{1}{2}L_{y}[K_{xHZ}(Z_{2}-Z_{5})+C_{xHZ}({\dot{Z}}_{2}-{\dot{Z}}_{5})]=0$$ (15)

$$J_{y}{\ddot{\theta }}_{y}+L_{H}[K_{xHZ}(Z_{1}-Z_{4})+C_{xHZ}({\dot{Z}}_{1}-{\dot{Z}}_{4})+K_{xHZ}(Z_{2}-Z_{5})+C_{xHZ}({\dot{Z}}_{2}-{\dot{Z}}_{5})]-L_{Q}[K_{xQZ}(Z_{3}-Z_{6})+C_{xQZ}({\dot{Z}}_{3}-{\dot{Z}}_{6})]=0$$ (16)

$$M_{1}{\ddot{Z}}_{4}+K_{xHZ}(Z_{4}-Z_{1})+C_{xHZ}({\dot{Z}}_{4}-{\dot{Z}}_{1})+K_{LHZ}(Z_{4}-Z_{7})+C_{LHZ}({\dot{Z}}_{4}-{\dot{Z}}_{7})=0$$ (17)

$$M_{2}{\ddot{Z}}_{5}+K_{xHZ}(Z_{5}-Z_{2})+C_{xHZ}({\dot{Z}}_{5}-{\dot{Z}}_{2})+K_{LHZ}(Z_{5}-Z_{8})+C_{LHZ}({\dot{Z}}_{5}-{\dot{Z}}_{8})=0$$ (18)

$$M_{3}{\ddot{Z}}_{6}+K_{xQZ}(Z_{6}-Z_{3})+C_{xQZ}({\dot{Z}}_{6}-{\dot{Z}}_{3})+K_{LQZ}(Z_{6}-Z_{9})+C_{LQZ}({\dot{Z}}_{6}-{\dot{Z}}_{9})=0$$ (19)

$$S_{ML}=Z_{0}+L_{Q}\theta_{y}-Z_{4}-\theta_{x}L_{y}/2$$ (20)

$$S_{MR}=Z_{0}+L_{Q}\theta_{y}-Z_{5}+\theta_{x}L_{y}/2$$ (21)

$$S_{N}=Z_{0}-L_{H}\theta_{y}-Z_{6}$$ (22)

$$Z_{1}=Z_{0}+\theta_{x}L_{y}/2+\theta_{y}L_{H}$$ (23)

$$Z_{2}=Z_{0}-\theta_{x}L_{y}/2+\theta_{y}L_{H}$$ (24)

$$Z_{3}=Z_{0}-\theta_{y}L_{Q}$$ (25)

where *K*_E_ is the kinetic energy of the aircraft system. *P*_E_ is the potential energy of the aircraft system. *D*_E_ is the energy consumption of the aircraft system. *M*_0_ is the mass of the aircraft. *M*_1_, *M*_2_, and *M*_3_ are the mass of left and right main landing gear and front landing gear, respectively. *Z*_0_ is the vertical displacement of aircraft affected by the international roughness index. *Z*_1_, *Z*_2_, and *Z*_3_ are the vertical displacement of left and right main landing gear and front landing gear, respectively. *J*_*x*_ and *J*_*y*_ are the mass moment of inertia of X and Y axis, respectively. *θ*_*x*_ and *θ*_*y*_ are the rolling rotation and pitching rotation, respectively. *K*_*xHZ*_ and *K*_*xQZ*_ are the suspension stiffness of left and right main landing gear and front landing gear, respectively. *C*_*xHZ*_ and *C*_*xQZ*_ are the suspension damping of left and right main landing gear and front landing gear, respectively. *K*_*LHZ*_ and *K*_*LQZ*_ are the tire stiffness of left and right main landing gear and front landing gear, respectively. *C*_*LHZ*_ and *C*_*LQZ*_ are the tire damping of left and right main landing gear and front landing gear, respectively. *Z*_4_, *Z*_5_, and *Z*_6_ are the tire vertical displacement of left and right main landing gear and front landing gear, respectively. *Z*_7_, *Z*_8_, and *Z*_9_ are the pavement roughness corresponding to left and right main landing gear and front landing gear, respectively. *L*_*H*_ and *L*_*Q*_ are the distance between center of gravity and the rear and front axle, respectively. *L*_*y*_ is the distance between left and right axle.

## 3. Numerical model

### 3.1 Establishing the numerical model

**Fig 2:**
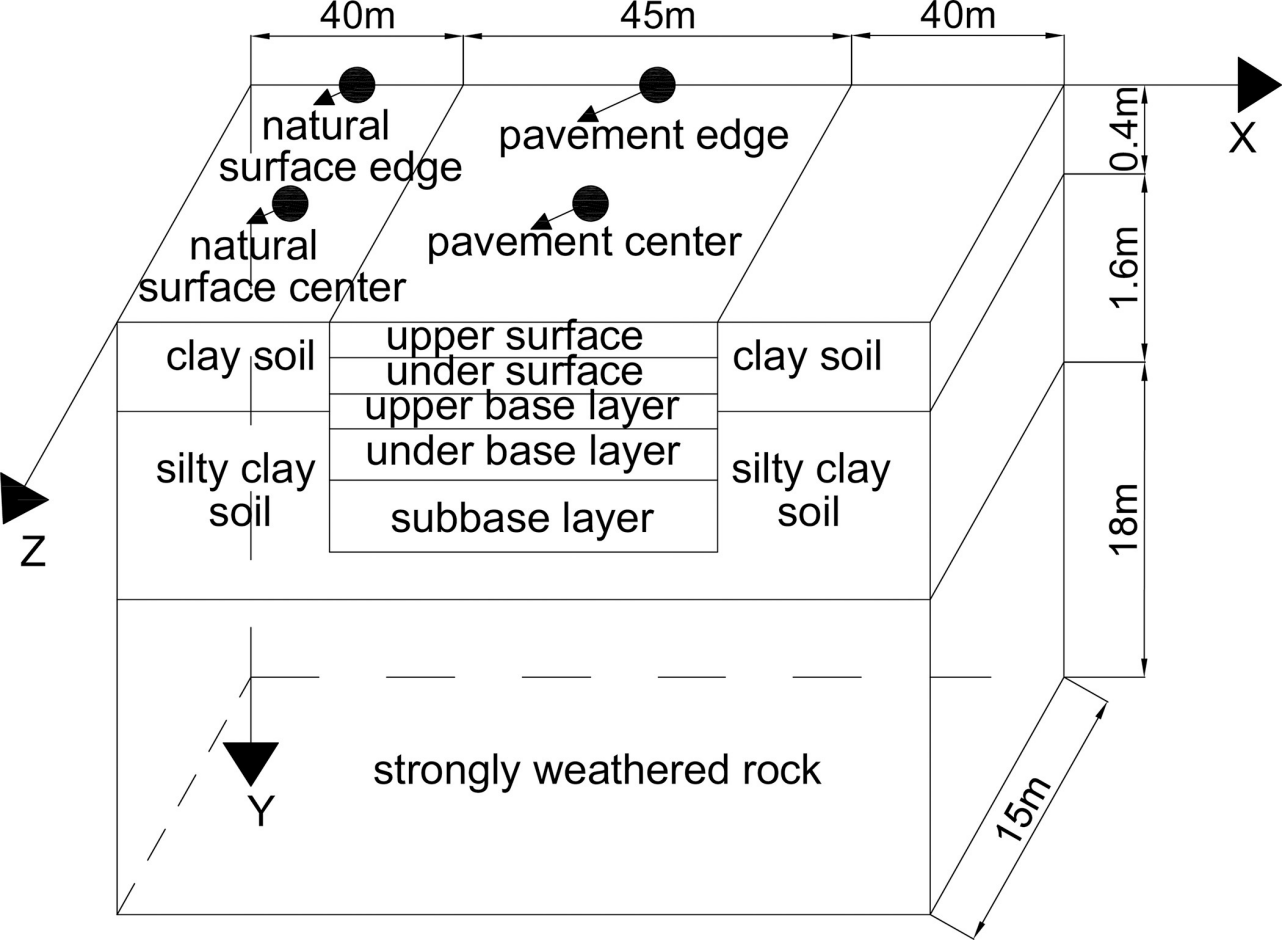
Schematic graph of numerical model.

In Fig 2, the natural surface was made of 0.4-m-thickness clay soil, 1.6-m-thickness silty clay soil, and 18-m-thickness strongly weathered rock based on an engineering geological survey [1, 26]. The specifications of the design of asphalt pavements for civil airports indicated that the pavement structure was made of upper and under surface, upper and under base layer, and subbase layer [26]. To eliminate the boundary effect, a 40-m-wide natural surface was placed on both sides of asphalt concrete pavements [26]. The numerical model of asphalt concrete pavement was 45-m-width and 15-m-length [26]. The depth was along the Y direction, width was along the X direction, and length was along the Z direction [26].

Eqs (8), (9) and (10) were applied to the boundary conditions of numerical models. The bottom of strongly weathered rock was constrained along the depth, length and width. The edges of strongly weathered rock, silty clay soil, and clay soil were denied the freedom degree of length and width direction [26]. The friction coefficient of interface layer was 0.5. The calculation time was 10 years [26]. The initial temperature field was -1°C [26, 30]. The phase change in a certain range of temperature was simulated by the apparent heat capacity method [26]. Tables 1 and 2 showed the thermal parameters of weathered rock, silty clay soil, clay soil, and pavement [1, 26]. The elastoplastic behavior of the soils was described by the Mohr-Coulomb constitutive model. Based on the research of Li [1] and Liu et al. [26], the mechanical parameters of permafrost were related to the temperature, and solved by Eqs (26)–(29). Table 3 showed the coefficients of expansion of strongly weathered rock, silty clay soil, and clay soil [1, 26]. Tables 4 and 5 showed the mechanical parameters of the pavement structure [1, 26].

$$E=a_{1}+b_{1}{\left|T\right|}^{m}$$ (26)

$$\upsilon =a_{2}+b_{2}|T|$$ (27)

$$c=a_{3}+b_{3}|T|$$ (28)

$$\theta =a_{4}+b_{4}|T|$$ (29)

where *E* and *ν* are the elastic modulus and the Poisson’s ratio, respectively. *c* and *θ* are the cohesion and the frictional angle, respectively. *a*_1_, *a*_2_, *a*_3_, *a*_4_, *b*_1_, *b*_2_, *b*_3_, and *b*_4_ are the regression coefficients of 28, 0.4, 0.15, 14, 27, -0.008, 0.09, and 0.6, respectively. When *T* is not less than zero, *b*_1_, *b*_2_, *b*_3_, and *b*_4_ are zero. *m* is the index of 0.6.

**Table 1:** Thermal parameters of strongly weathered rock, silty clay soil, and clay soil.

Thermal parameter		-20°C	-10°C	-5°C	-2°C	-1°C	-0.5°C	0°C	20°C
Clay soil	Density/(kg/m^3^)	1870	1870	1870	1870	1870	1870	1870	1870
	Thermal conductivity /(W/(m·°C))	2.4	2.4	2.4	2.4	2.4	2.4	1.54	1.54
	Heat capacity/(J/(kg·°C))	835	840	850	860	870	900	1070	1070
Silty clay soil	Density/(kg/m^3^)	1950	1950	1950	1950	1950	1950	1950	1950
	Thermal conductivity /(W/(m·°C))	1.81	1.81	1.81	1.81	1.81	1.81	1.5	1.5
	Heat capacity/(J/(kg·°C))	970	1050	1090	1115	1140	1210	1285	1285
Strongly weathered rock	Density/(kg/m^3^)	2150	2150	2150	2150	2150	2150	2150	2150
	Thermal conductivity /(W/(m·°C))	2.5	2.5	2.5	2.5	2.5	2.5	2.01	2.01
	Heat capacity/(J/(kg·°C))	950	1060	1110	1140	1190	1250	1350	1350

**Table 2:** Thermal parameters of the pavement structure.

Pavement structure	Material	Density/(kg/m^3^)	Thickness/m	Heat capacity/(J/(kg·°C))	Thermal conductivity/(W/(m·°C))
Upper surface	Asphalt concrete	2300	0.15	1670	1.15
Under surface	Asphalt concrete	2320	0.15	1670	1.20
Upper base layer	Cement-stabilized macadam	2200	0.3	960	1.56
Under base layer	Cement-stabilized macadam	2233	0.35	920	2.04
Subbase layer	Graded macadam	2000	0.5	1100	1.68

**Table 3:** Expansion coefficients of strongly weathered rock, silty clay soil, and clay soil.

Temperature/°C	Clay soil	Silty clay soil	Strongly weathered rock
-30	-5.33×10^−5^	-6.08×10^−5^	-8.55×10^−5^
-20	-1.22×10^−4^	-1.38×10^−4^	-1.22×10^−4^
-18	-1.39×10^−4^	-1.61×10^−4^	-1.43×10^−4^
-10	-3.26×10^−4^	-3.66×10^−4^	-3.18×10^−4^
-5	-1.38×10^−3^	-2.04×10^−3^	-1.26×10^−3^

The expansion coefficient is a dimensionless index.

**Table 4:** Mechanical parameters of the pavement structure.

Pavement structure	Material	Elastic modulus/MPa	Poisson’s ratio	Expansion coefficient
Upper surface	Asphalt concrete	Table 5	0.25	$\beta =2.496e^{0.01467T}\times 10^{-5}$
Under surface	Asphalt concrete	Table 5	0.25	$\beta =2.496e^{0.01467T}\times 10^{-5}$
Upper base layer	Cement-stabilized macadam	3800	0.25	3.80×10^−5^
Under base layer	Cement-stabilized macadam	3400	0.25	3.40×10^−5^
Subbase layer	Graded macadam	175	0.35	1.75×10^−4^

*β* is the expansion coefficient. Poisson’s ratio and expansion coefficient are both the dimensionless index.

**Table 5:** Elastic modulus of the surface layer.

Temperature/°C	-30	-20	-10	0	10	20	30
Elastic modulus/MPa	8046	5011	3160	1986	1021	784	452

The three-dimensional mass-spring-damper system with six degrees of freedom (DOFs) was applied to simulate the aircraft, as shown in Fig 1 and Table 6 [31]. The body of aircraft was three DOFs, such as the vertical displacement at the mass center, rolling rotation and pitching rotation. Each tire was a lumped mass with vertical displacement. The aircraft was described by beam elements (MPC184). The suspension and nonsuspension systems and the inertia moments of pitching and rolling rotations were described by mass elements (Mass21). The spring and damper were described by a spring-damper element (Combine14). The dynamic load of B737-800 aircraft was described as a uniformly distributed rectangular load, and arranged symmetrically along the centerline of the upper surface. The elements of the loading area were selected at 0.30-m-width and 0.43-m-length according to the widths of tire ribs and grooves [32]. The tire pressure was calculated by the loading area and tire load. The moving load was described by controlling the loading area on pavement surface and the specific interval based on the speed. The speed of aircraft was from 10m/s to 70m/s in the numerical model.

**Table 6:** Parameters of aircraft.

Measurement	Parameter	Measurement	Parameter
*M*_0_/kN	386	*C*_*xQZ*_/(kN/(m∙s))	5
*J*_*x*_/(kN∙m^2^)	20000	*K*_*LHZ*_/(kN/m)	9600
*J*_*y*_/(kN∙m^2^)	7200	*K*_*LQZ*_/(kN/m)	2400
*M*_1_, *M*_2_/kN	74.55	*C*_*LHZ*_/(kN/(m∙s))	24
*M*_3_/kN	27.85	*C*_*LQZ*_/(kN/(m∙s))	6
*K*_*xHZ*_/(kN/m)	4800	*L*_*H*_/m	0.78
*K*_*xQZ*_/(kN/m)	1200	*L*_*Q*_/m	14.82
*C*_*xHZ*_/(kN/(m∙s))	20	*L*_*y*_/m	5.7

### 3.2 Verifying the numerical model

**Fig 3:**
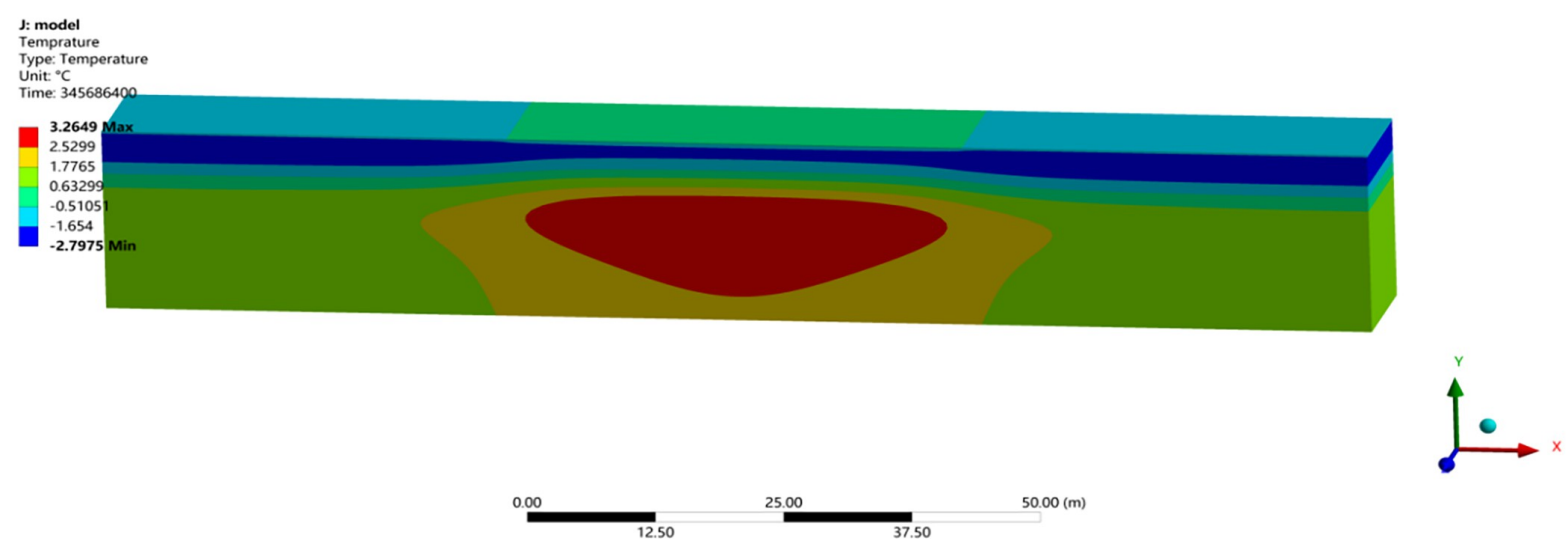
Temperature field after 10 years of operation in the northeast of China.

**Fig 4:**
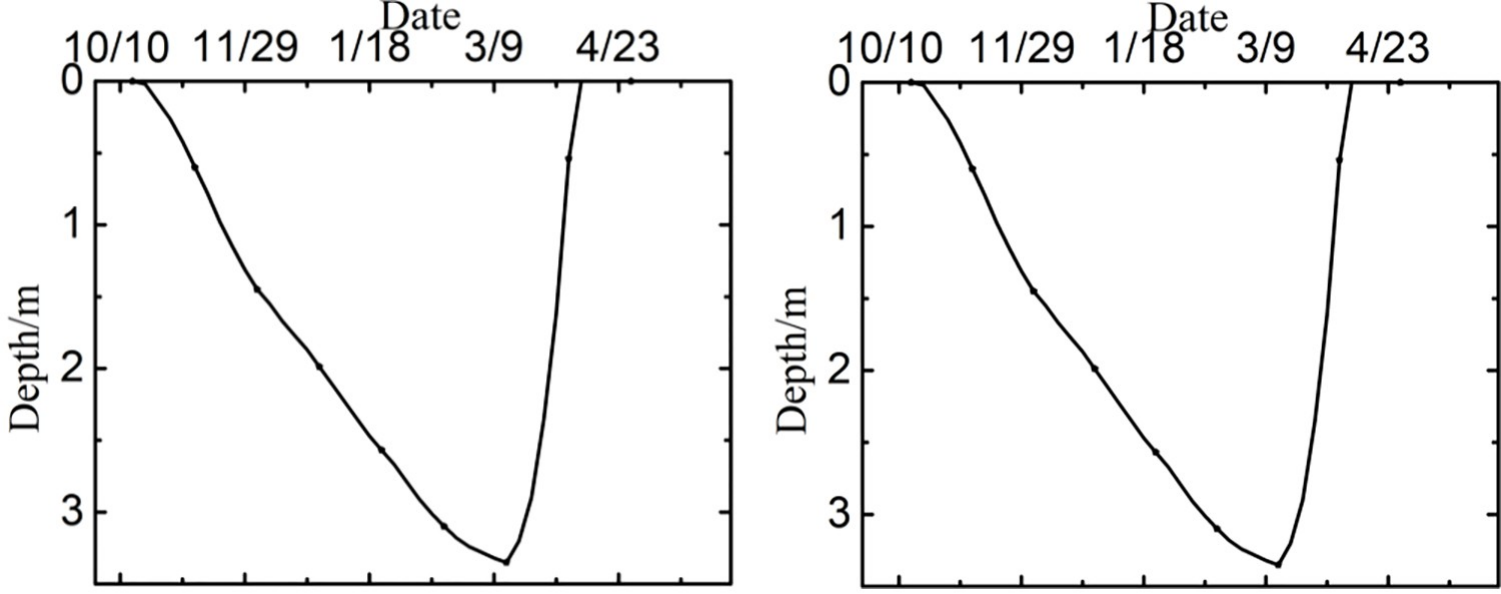
The depth of frozen layer after 10 years of operation at (a) the center of the pavement, and (b) the edge of the pavement.

**Fig 5:**
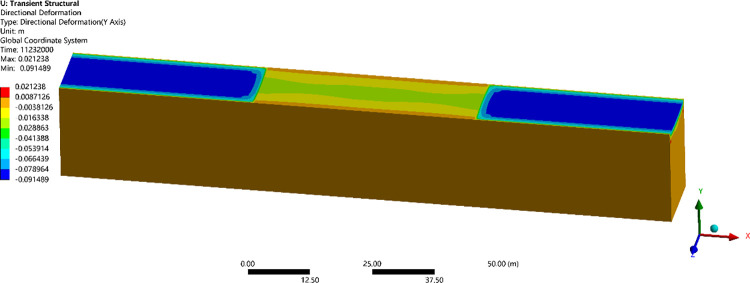
Displacement field in the northeast of China after one year of operation.

Owing to a lack of testing data on runways, the reliability of numerical model was verified by a comparison with the temperature field, frozen layer depth, and settlement in previously studies. A transient thermal analysis of runway was conducted over 10 years with the initial temperature field. Figs 3 and 4 showed the temperature field and the depth of frozen layer after 10 years of operation [26]. Fig 5 showed the displacement field after one year of operation [26].

Fig 3 showed that the maximum depth of the frozen layer was on the 351^th^ day, at the end of March in the second year, which were in agreement with the study of Liu et al. [33], Wang [34] and Liu et al. [26]. Fig 4 showed that the pavement started to freeze in the middle of October, reached the maximum depth at the end of March, began to thaw at the beginning of April, and had completely thawed by the end of April, which were consistent with the report from Li [1], Duan [30] and Liu et al. [26]. Moreover, the maximum depth of the frozen layer was about 3.35m in runway engineering and deeper than that of highway and railway engineering, which was consistent with the research of Bjella [35], Simon and Dilley [36] and Liu et al. [26]. Fig 5 showed that the maximum settlement was about on the 130^th^ day, at the middle of August. The maximum settlements at the center of the pavement was -0.023m, and the maximum settlements at the center of the natural surface was -0.091m in runway engineering, which were in accord with the result from LeBlanc et al. [37] and Liu et al. [26]. Hence, the parameters and boundary conditions of runway models were reasonably applied to analyze the settlement response of runway in a permafrost region.

## 4. Results and discussion

### 4.1 Evolution of settlement

**Fig 6:**
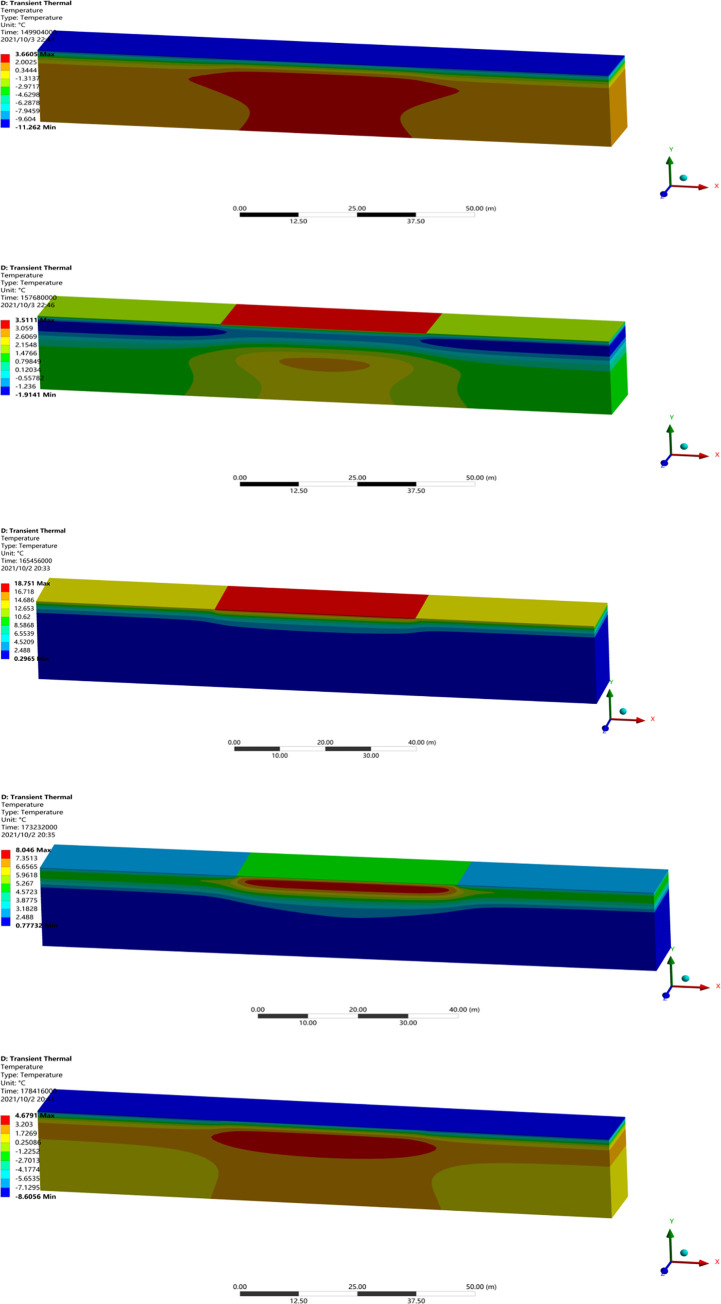
Temperature field nephograms on (a) January 1st, (b) April 1st, (c) July 1st, (d) October 1st, and (e) December 1st.

To observe the evolution of settlement, the temperature field nephograms on the first day of each month in the first year were extracted in Fig 6.

In Fig 6(A), January was the coldest month and the surface of the pavement was significantly influenced by the external environment and began to cool down firstly. Hence, the temperature of the pavement increased with the depth increased, and was barely effected by the external environment when the depths were more than 12m. In Fig 6(B), the air temperature increased from February to April. In April, the temperature of the pavement center exhibited high-low-high-low characteristics with the increase of the depth. The surface of the pavement began to warm up firstly with the growth of the air temperature. The temperature of the pavement changed more and more slowly with the increase of the depth, and the high-temperature interlayer became smaller gradually in the pavement structure. In Fig 6(C), the air temperature continued to increase from May to July. As July was the hottest month, the temperature of the pavement center exhibited high-low characteristics with the growth of the depth. The high-temperature interlayer disappeared as the whole pavement was in high-temperature. In Fig 6(D), the air temperature gradually decreased from August to October. In October, the temperature of the pavement center started to slow and exhibited low-high-low characteristics with the increase of the depth. The surface of the pavement was effected by the external environment firstly, and began to slow in temperature. The temperature of the pavement became slower as the depth increased, and the high-temperature interlayer could be clearly seen in the pavement structure. In Fig 6(E), because the air temperature continued to decrease from November to December, the cooling depth of the pavement increased and the high-temperature interlayer was deepening and expanding.

### 4.2 Settlement under different loads

**Fig 7:**
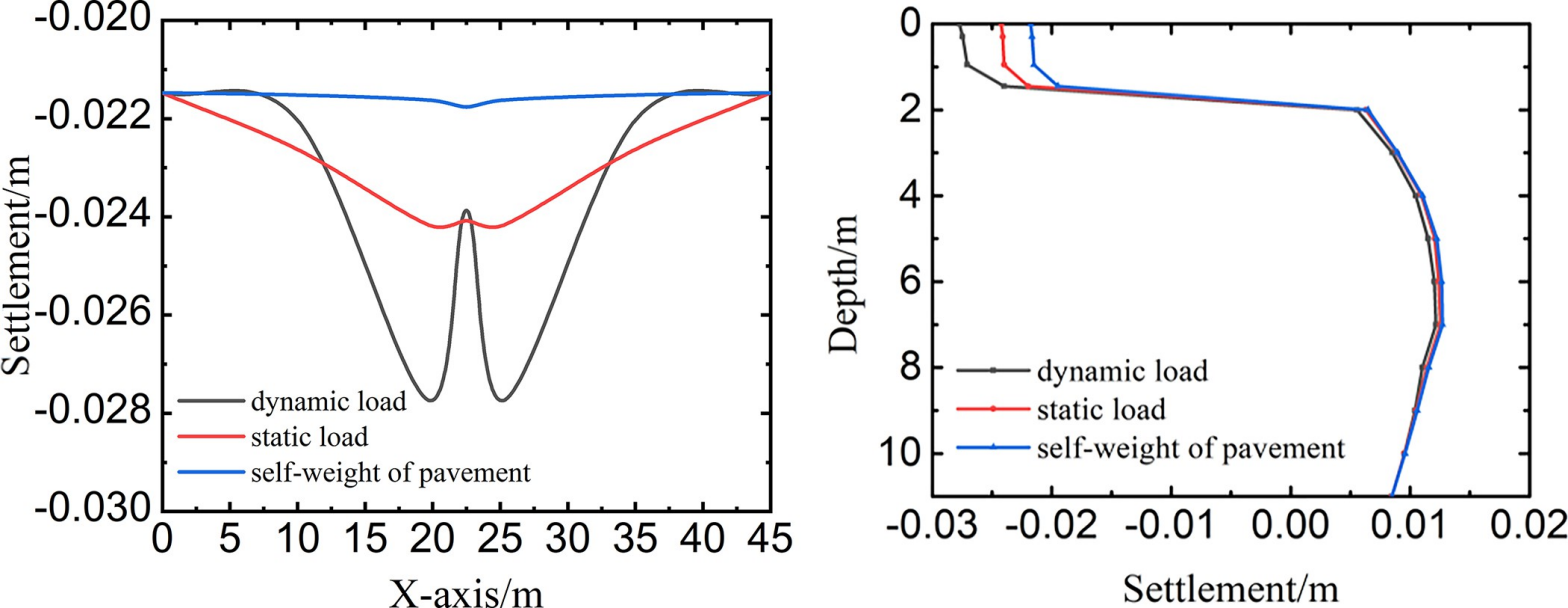
Influence of different loads on the settlement along (a) X-axis, and (b) depth.

To observe the influence of different loads on the settlement, the settlements under different loads along X-axis and depth at the middle of August of the tenth year were shown in Fig 7. In Fig 7, the dynamic load of aircraft was obtained at the international roughness index of 3, the taxiing speed of 30m/s, and the aircraft’s weight of 57878kg.

In Fig 7(A), the settlement of self-weight of pavement was the smallest. The settlement of dynamic load was larger than that of static load. Because the settlement of permafrost was mainly influenced by temperature and load, which was a coupling process of temperature and load. When the warm season came, the frozen ice in the soil thawed into water to reduce the volume and strength of soil. The permafrost was subject to settlement because of the deadweight of the pavement structure and subgrade. Meanwhile, the load of aircraft also caused the settlement of the permafrost. Moreover, because the dynamic load included the self-weight of aircraft and the additional load caused by the international roughness index and taxiing speed, the dynamic load of aircraft was greater than that of static load. And the settlement of dynamic load was larger than that of static load. In Fig 7(B), the shape of settlement along the depth was similar at different loads. Firstly, the settlement decreased slowly at the depth of 0m to 1.45m, then decreased quickly at the depth of 1.45m to 2m, finally tended to be stable at the depth of 2m to 11m. When the depth was from 0m to 1.45m, the settlement of dynamic load was the largest and the settlement of static load was bigger than that of self-weight of pavement. When the depth was from 2m to 11m, the difference of settlement was little caused by dynamic load, static load, and self-weight of pavement. Because the elastic modulus of permafrost subgrade varied greatly because the temperature caused the settlement in a relatively shallow range. Meanwhile, the stress induced by the load decreased gradually along the depth direction, so the settlement caused by the pavement structure and aircraft also reduced gradually along the depth direction.

### 4.3 Influence of different parameters on the settlement

In the aircraft and pavement response analysis of numerical models, the international roughness index of 1 was to represent a new pavement or the best pavement condition, the international roughness index of 3 was to represent the good pavement condition with a little damage, and the international roughness index of 5 was to describe the poor pavement condition with much damage [29]. The speed of 10m/s indicated that the aircraft was beginning to taxiing, the speed of 30m/s indicated an accelerated taxiing of aircraft, and the speed of 70m/s indicated that the plane was about to take off. The weight of 42275kg was the weight of aircraft at no load, the weight of 57878kg was the weight of aircraft at some load, and the weight of 73482kg was the weight of aircraft at full load.

**Fig 8:**
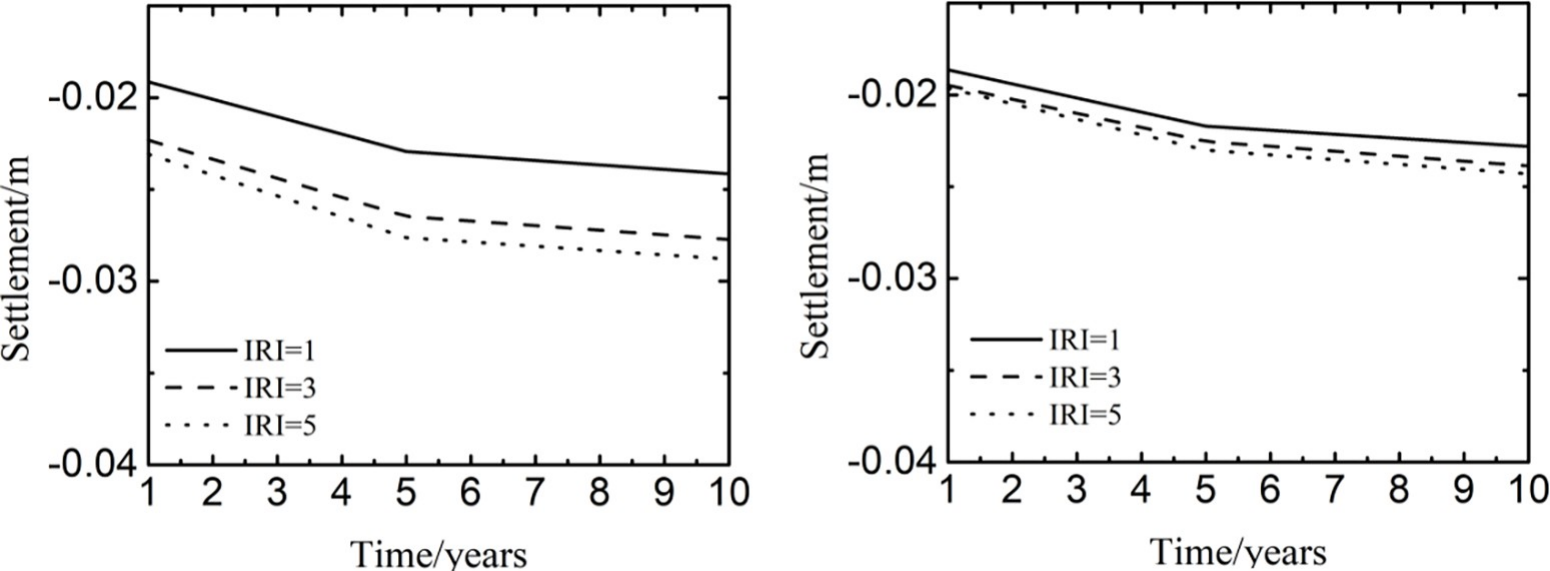
Influence of international roughness index at the center of (a) the wheel track and (b) the pavement.

Fig 8 represented the time history curve of settlement at different positions of pavement when the taxiing speed was 30m/s and the weight was 57878kg. In Fig 8(A), when the international roughness index was from 1 to 5, the shapes of time history curves were similar with different peaks of time history curves. When the time was from 1^st^ year to 5^th^ year, the settlement had a rapid increase. While the time was from 5^st^ year to 10^th^ year, the settlement had a minimal change. The reason might be that the consolidation settlement of soil mainly occurred in the previous years, and the subsequent consolidation settlement gradually decreased. Meanwhile, the voids and moisture in the soil decreased gradually to reduce the settlement of soil. When the international roughness index varied from 1 to 3 at the same time, the settlement increased quickly. While the international roughness index varied from 3 to 5 at the same time, the settlement grew little. It was because that the dynamic load of aircraft caused by damaged pavement was much greater than that caused by good pavement. In Fig 8(B), the development laws of time history curves were similar to those of Fig 8(A), and the peaks of time history curves were smaller than those of Fig 8(A). The explanation of the phenomenon was that the load at the center of the wheel track was greater than that at the center of the pavement, and the settlement at the center of the wheel track was larger than that at the center of the pavement.

**Fig 9:**
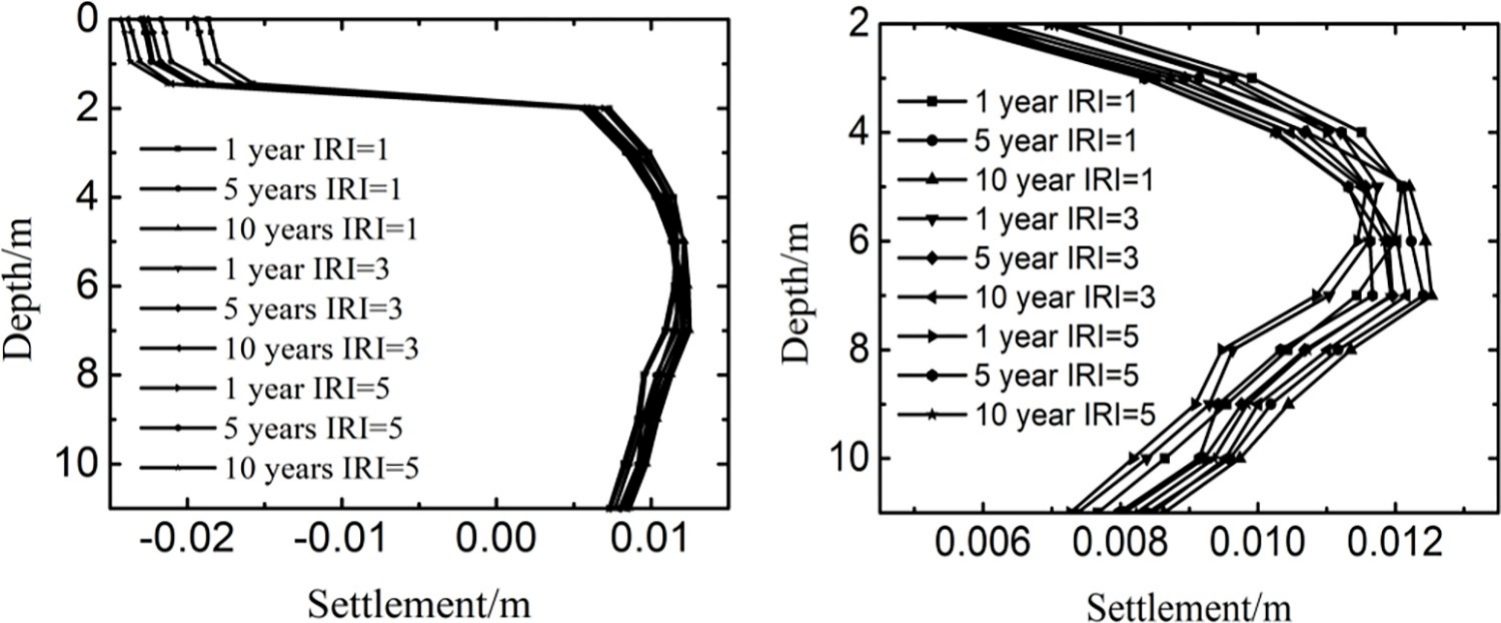
Influence of international roughness index on the settlement at (a) 0m-11m and (b) 2m-11m.

The top of the upper surface layer was set to 0m. Fig 9(A) showed the influence of international roughness index and years on the settlement at the depth of 0m-11m. Fig 9(B) showed the influence of international roughness index and years on the settlement at the depth of 2m-11m. Fig 9(B) was an enlarged view of Fig 9(A) at the depth of 2m-11m. As the time and the international roughness index varied, the change law of settlement was similar along the depth. Firstly, the settlement decreased little with the depth being from 0m to 1.45m, then changed large with the depth being from 1.45m to 2m, and finally tended to fluctuate between 0.0055m and 0.0125m with the depth being from 2m to 11m. The phenomenon showed that the settlement mainly appeared in the soil instead of the pavement structure layers, and the significant area of settlement was below the pavement structure layers. Therefore, protecting the soil temperature stability below the pavement structure layers was helpful to control the settlement of runway.

**Fig 10:**
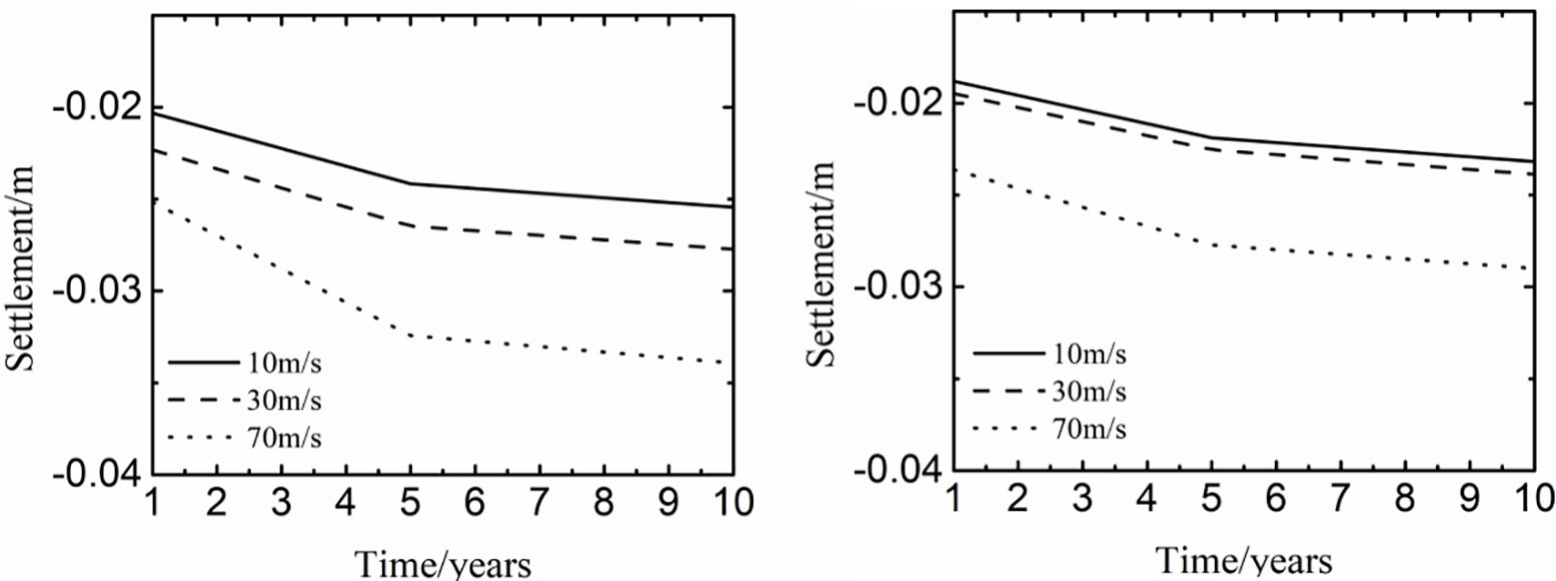
Influence of taxiing speed at the center of (a) the wheel track and (b) the pavement.

Fig 10 described the time history curve of settlement at different positions of pavement when the international roughness index was 3 and the weight was 57878kg. In Fig 10(A), the change laws of time history curves were similar with different peaks of time history curves, which were different with the increase of the taxiing speed. Because the reduction of voids and moisture in the soil mainly occurred in the previous years and the subsequent reduction was small and slow, the settlement after 5 years of operation in the northeast of China varied little at different taxiing speed. Moreover, because the taxiing speed was beneficial to increase the dynamic load of aircraft, the settlement grew with the increase of the taxiing speed. In Fig 10(B), the shape of time history curves were the same to those of Fig 10(A), and the peaks of time history curves were smaller than those of Fig 10(A). The reason might be that the settlement caused by the wheel at the center of the wheel track was greater than that at the center of the pavement. The varied value of settlement at the taxiing speed of 10m/s to 30m/s was significantly smaller than that at the taxiing speed of 30m/s to 70m/s. The phenomenon indicated that the faster the taxiing speed, the larger the settlement caused by the dynamic load of aircraft.

**Fig 11:**
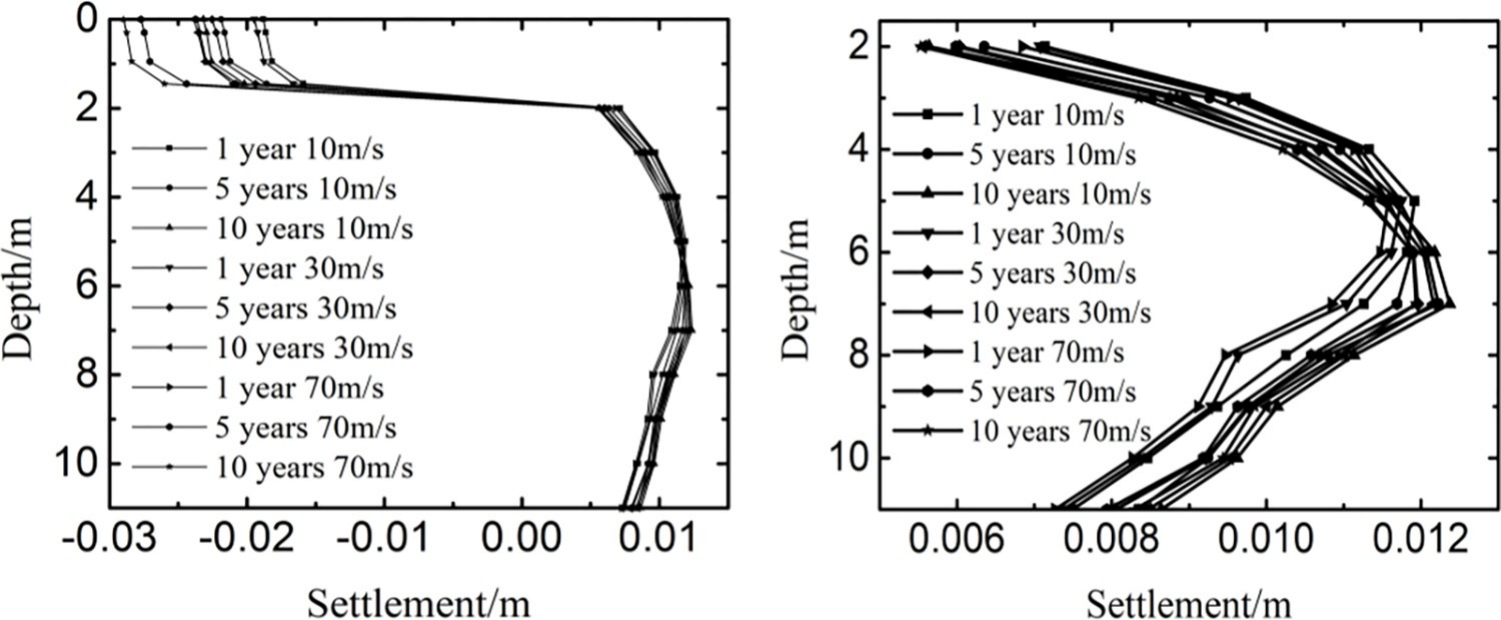
Influence of taxiing speed on the settlement at (a) 0m-11m and (b) 2m-11m.

Fig 11 showed the influence of taxiing speed on the settlement at different years and depth. As the time and the taxiing speed varied, the change law of settlement was similar along the depth. The settlement had little change with the depth being from 0m to 1.45m, varied largely with the depth being from 1.45m to 2m, and kept stable in a small range with the depth being from 2m to 11m. Because the thickness of the pavement structure layer was 1.45m, and the settlement mainly appeared in the subgrade and decreased as the depth increased. Hence, the temperature stability of soil in a certain range below the pavement structure was the key to control the settlement of runway.

**Fig 12:**
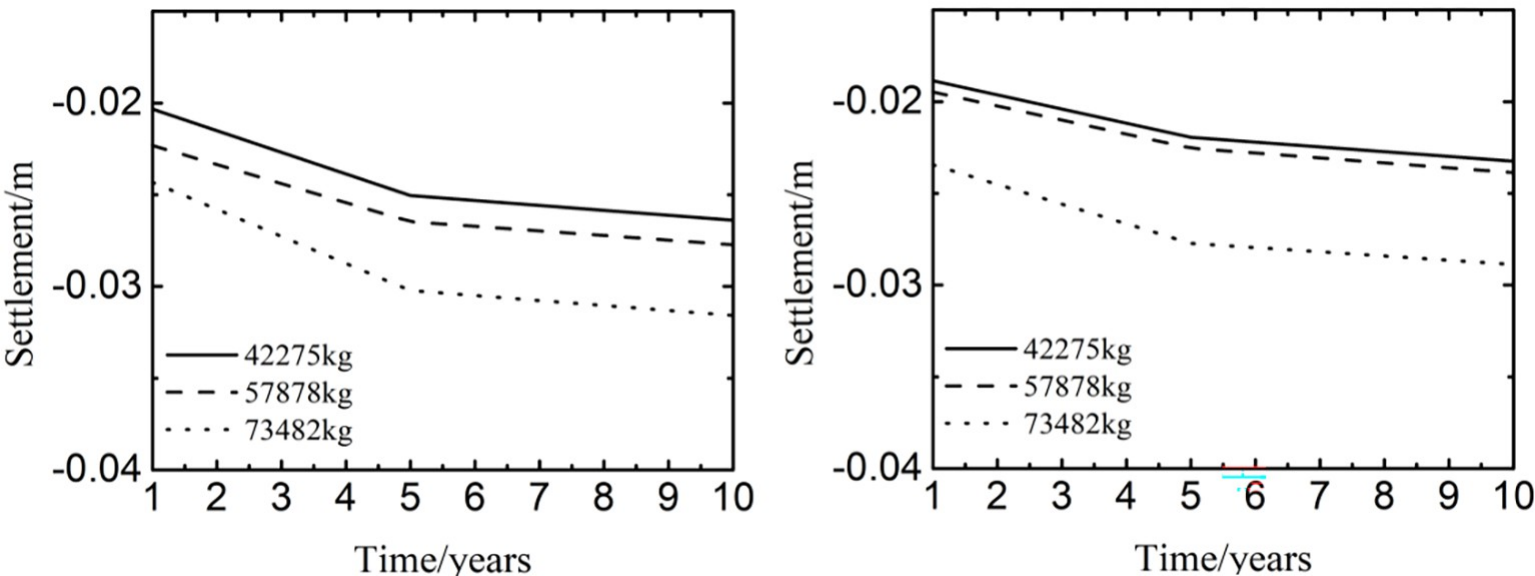
Influence of aircraft’s weight at the center of (a) the wheel track and (b) the pavement.

Fig 12 represented the time history curve of settlement at different positions of pavement when international roughness index was 3 and the taxiing speed was 30m/s. In Fig 12(A), time history curves of settlements had the similar shapes and different peaks with the growth of aircraft’s weight. The settlement was positively correlated with the aircraft’s weight. When the aircraft’s weight varied from 42275kg to 73482kg, the settlement tended to be stable after 5 years of operation in the northeast of China. Moreover, the settlement was more than 3cm at the aircraft’s weight of 73482kg after 5 years of operation in the northeast of China. In Fig 12(B), the laws of time history curves were similar and the peaks of time history curves were different with the aircraft’s weight being from 42275kg to 73482kg, which was similar to those of Fig 12(A). However, the settlement was smaller than that of Fig 12(A) at the same time and aircraft’s weight. The reason was that the load of aircraft decreased with the growth of distance away from the wheel track, and the settlement was positively correlated with the aircraft’s weight.

**Fig 13:**
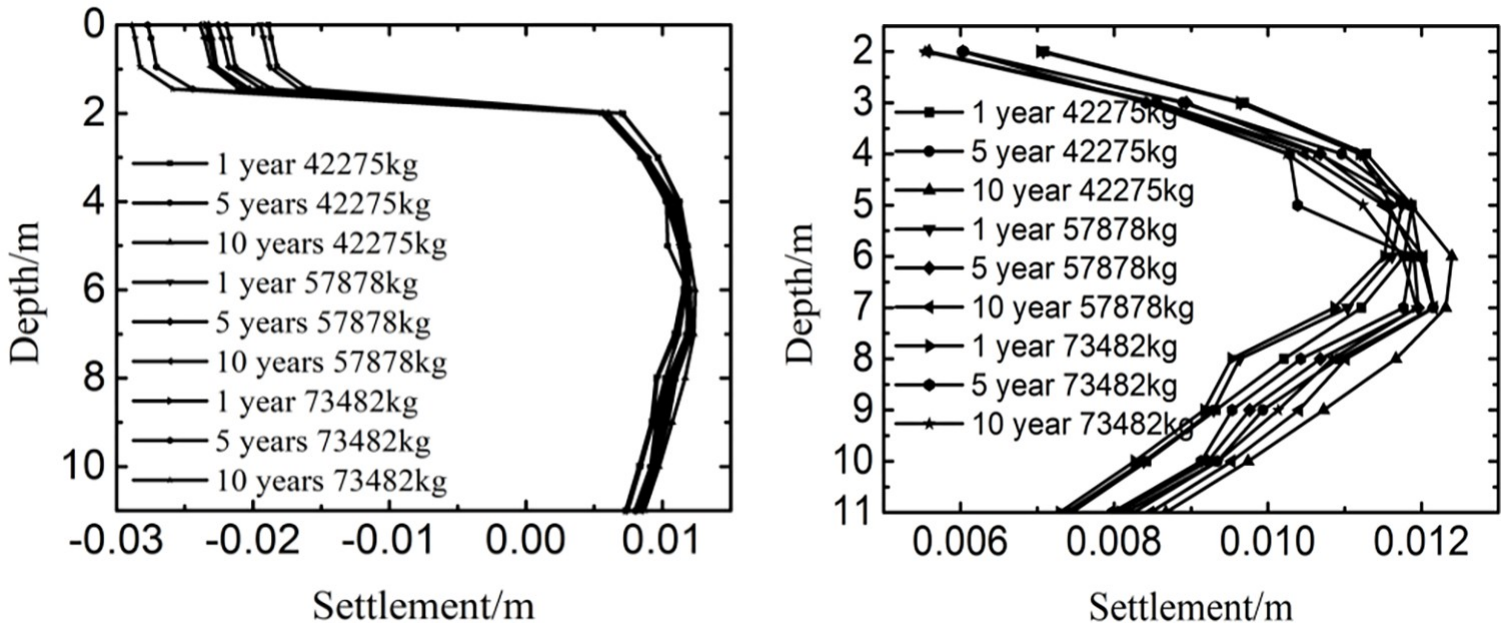
Influence of aircraft’s weight on the settlement at (a) 0m-11m and (b) 2m-11m.

Fig 13 showed the influence of aircraft’s weight on the settlement at different years and depth. when the time and the aircraft’s weight increased, the settlement decreased slowly with the depth being from 0m to 1.45m, reduced quickly with the depth being from 1.45m to 2m, and tended to be the range of 0.0055m to 0.0125m with the depth being from 2m to 11m. Because the material of pavement structure layer had less voids and moisture than that of subgrade, and the void and moisture were helpful to cause the settlement of runway. Hence, controlling the void and moisture was also beneficial to keep the stability of subgrade in a permafrost region.

## 5. Conclusions

This study proposed and verified a numerical model of asphalt concrete pavement and subgrade in permafrost regions by the temperature field, frozen layer depth, and settlement comparison of previous research without considering the dynamic load of aircraft. This study also analyzed the settlement under different parameters. The main conclusions were shown as follows:

1. In January, the temperature of pavement decreased as the depth increased, and was barely effected by the external environment when the depths were more than 12m. In April, the temperature of pavement center exhibited high-low characteristics with the growth of the depth. The surface of pavement began to warm firstly as the air temperature increased. The temperature of pavement slowed with the increasing depth, and an obvious low-temperature interlayer appeared in the pavement structure. In July, the temperature of pavement center exhibited high-low characteristics with the depth increased. The low-temperature interlayer disappeared. In October, the temperature of pavement center started to slow and exhibited low-high-low characteristics with the depth increased. The temperature of pavement became slower with the increase of the depth, and the high-temperature interlayer could be seen in the pavement. When it was from November to December, the cooling depth of pavement increased and the high-temperature interlayer was deepening and expanding.
2. The shapes of settlements under self-weight of pavement, static load, and dynamic load were similar and reduced gradually along the depth direction. The settlement of static load was larger than that of self-weight of pavement, and smaller than that of dynamic load. The settlements of different loads decreased slowly at the depth of 0m to 1.45m, then decreased quickly at the depth of 1.45m to 2m, finally tended to be stable at the depth of 2m to 11m.
3. When the time was from 1st year to 5th year, the settlement had a rapid increase. While the time was from fifth year to tenth year, the settlement had a minimal change. The settlement at the center of the wheel track was larger than that at the pavement center. The laws of time history curves were similar with different peaks of time history curves for different international roughness index, taxiing speed, and aircraft’s weight. As the time varied, the change law of settlement was similar along the depth for different international roughness index, taxiing speed, and aircraft’s weight. Firstly, the settlement decreased little with the depth being from 0m to 1.45m, then changed large with the depth being from 1.45m to 2m, and finally tended to fluctuate between 0.0055m and 0.0125m with the depth being from 2m to 11m.
4. When the international roughness index varied from 1 to 3 at the same time, the settlement increased quickly. While the international roughness index varied from 3 to 5 at the same time, the settlement grew little. The varied value of settlement at the taxiing speed of 10m/s to 30m/s was significantly smaller than that at the taxiing speed of 30m/s to 70m/s. When the aircraft’s weight varied from 42275kg to 73482kg, the settlement increased in the first five years and tended to be stable after 5 years of operation in the northeast of China. Moreover, the settlement was more than 3cm at the aircraft’s weight of 73482kg after 5 years of operation in the northeast of China.
